# Apolipoprotein E Alleles and Motor Signs in Older Adults with Alzheimer’s Dementia

**DOI:** 10.3390/ijms26178562

**Published:** 2025-09-03

**Authors:** Ioannis Liampas, Silvia Demiri, Vasileios Siokas, Antonia Tsika, Chrysa Marogianni, Polyxeni Stamati, Grigorios Nasios, Lambros Messinis, Constantine G. Lyketsos, Efthimios Dardiotis

**Affiliations:** 1 Department of Neurology, University Hospital of Larissa, School of Medicine, University of Thessaly, 41100 Larissa, Greece; antonellatsi@hotmail.com (A.T.); c.marogianni@gmail.com (C.M.); tzeni_0@yahoo.gr (P.S.); edar@med.uth.gr (E.D.); 2 School of Medicine, University of Patras, 26504 Patras, Greece; sylviantemiri@gmail.com; 3 Department of Speech and Language Therapy, School of Health Sciences, University of Ioannina, 45500 Ioannina, Greece; grigoriosnasios@gmail.com; 4 Laboratory of Neuropsychology and Behavioural Neuroscience, School of Psychology, Aristotle University of Thessaloniki, 54124 Thessaloniki, Greece; lmessinis@psy.auth.gr; 5 Department of Psychiatry and Behavioral Sciences, Johns Hopkins School of Medicine, Baltimore, MD 21205, USA; kostas@jhmi.edu

**Keywords:** Alzheimer’s dementia, *apolipoprotein E*, motor signs, tremor, rigidity, bradykinesia, posture, gait

## Abstract

We investigated associations between *apolipoprotein E* (*APOE*) alleles and motor manifestations in Alzheimer’s dementia (AD) capitalizing on National Alzheimer’s Coordinating Center data: the baseline evaluations of older adults (≥60 years) with a diagnosis of AD were analyzed. Those with a concomitant diagnosis Parkinson’s disease or other parkinsonian syndrome, and those treated with anti-parkinsonian agents were excluded. Three *APOE* groups were formed: *APOE2* (*APOE2* carriers), *APOE3* (*APOE3/APOE3*) and *APOE4* (*APOE4/APOE4, APOE4/APOE3*). UPDRS-III was used to assess the presence or absence of motor signs in 9 domains. Adjusted binary logistic models featuring the three *APOE* groups as exposures and motor domains as outcomes were estimated. There were 389 individuals in the *APOE2*, 1799 in the *APOE3* and 2791 in the *APOE4* groups. Compared to the *APOE2* group, individuals in the APOE4 group had lower odds of having at least one motor sign [0.64 (0.50–0.82)]. Among motor signs, rigidity [0.53 (0.34–0.81)], bradykinesia [0.56 (0.40–0.77)], impaired chair rise [0.54 (0.37–0.78)] and impaired posture-gait [0.54 (0.36, 0.81)] exhibited significant associations. Exploratory analyses featuring *APOE* genotypes suggested dose–response relationships for both *APOE2* and *APOE4*. In conclusion, *APOE2* confers a risk towards motor (mainly parkinsonian) signs in AD. *APOE4* may have a protective effect.

## 1. Introduction

The *apolipoprotein E* (*APOE*) gene, highly expressed in astrocytes, encodes a key component of lipoprotein particles. APOE is a multifunctional protein, involved in different homeostatic processes of the central nervous system; lipid transport, glucose metabolism, synaptic plasticity, neuroinflammation and vascular integrity [1,2,3]. *APOE* has three major allelic variants *APOE2* (7–10%), *APOE3* (75–83%), and *APOE4* (7–16% of the general population), which differ by single amino acid substitutions at positions 112 and 158 [4]. Individuals inherit one copy of the gene from each parent, resulting in six possible genotypes, with *APOE3/APOE3* being the most common [5].

*APOE* was the first genetic locus associated with the risk of late-onset Alzheimer’s disease dementia (AD) in 1993 [6]. Carriers of a single *APOE4* allele have a 2 to 3-fold increased risk for AD, while those with two copies have a 10 to 15-fold greater risk [4]. Furthermore, *APOE4* is related to an earlier age of AD onset [7,8]. On the other hand, *APOE2* is linked to a lower risk of incident AD and a later age of disease onset [9,10]. Of note, across the AD continuum, *APOE2* has been linked to less prominent AD-related pathological alterations, more severe non-AD neuropathology, and atypical, non-amnestic AD phenotypes [11,12,13,14]. Recently, *APOE2* was associated with more severe pathology in primary tauopathies such as progressive supranuclear palsy and corticobasal degeneration, supporting an association between *APOE2* and non-AD pathologies [9,15].

*APOE* is also implicated in the pathogenesis of α-synucleinopathies [16]. *APOE4* carriage increases the risk of Lewy Body Dementia, both Dementia with Lewy bodies and Parkinson’s Disease (PD) Dementia [16]. In contrast, research in PD established the following associations: meta-analyses support an overall risk-conferring effect of *APOE2* towards PD [17,18,19], with some studies suggesting the presence of several additional, more complex, ethnic and/or genotypic associations [19,20]. In particular, APOE2/APOE4 has been found to confer a substantial risk towards PD in Asians, and APOE3/APOE4 to modestly increase the risk for PD in Latin-Americans [16].

Parkinsonian signs are present in AD with increasing frequency as the disease progresses [21]. Bradykinesia, rigidity and postural/gait disturbances are most commonly observed [21,22,23,24]. However, the determinants of motor signs in AD remain largely unknown. We investigated associations between *APOE* genotype and motor manifestations in AD. Based on the initial reports of the risk-conferring properties of *APOE2* towards PD, as well as the neuropathologic and atypical phenotypic associations of *APOE2* across the AD continuum, we hypothesized that *APOE2* alleles will increase the odds of having motor sings in AD. We capitalized on data from the Uniform Data Set (UDS), a central repository of data, stewarded by the National Alzheimer’s Coordinating Center (NACC) [25].

## 2. Results

### 2.1. Participant Characteristics and Missing Data

**Figure 1:**
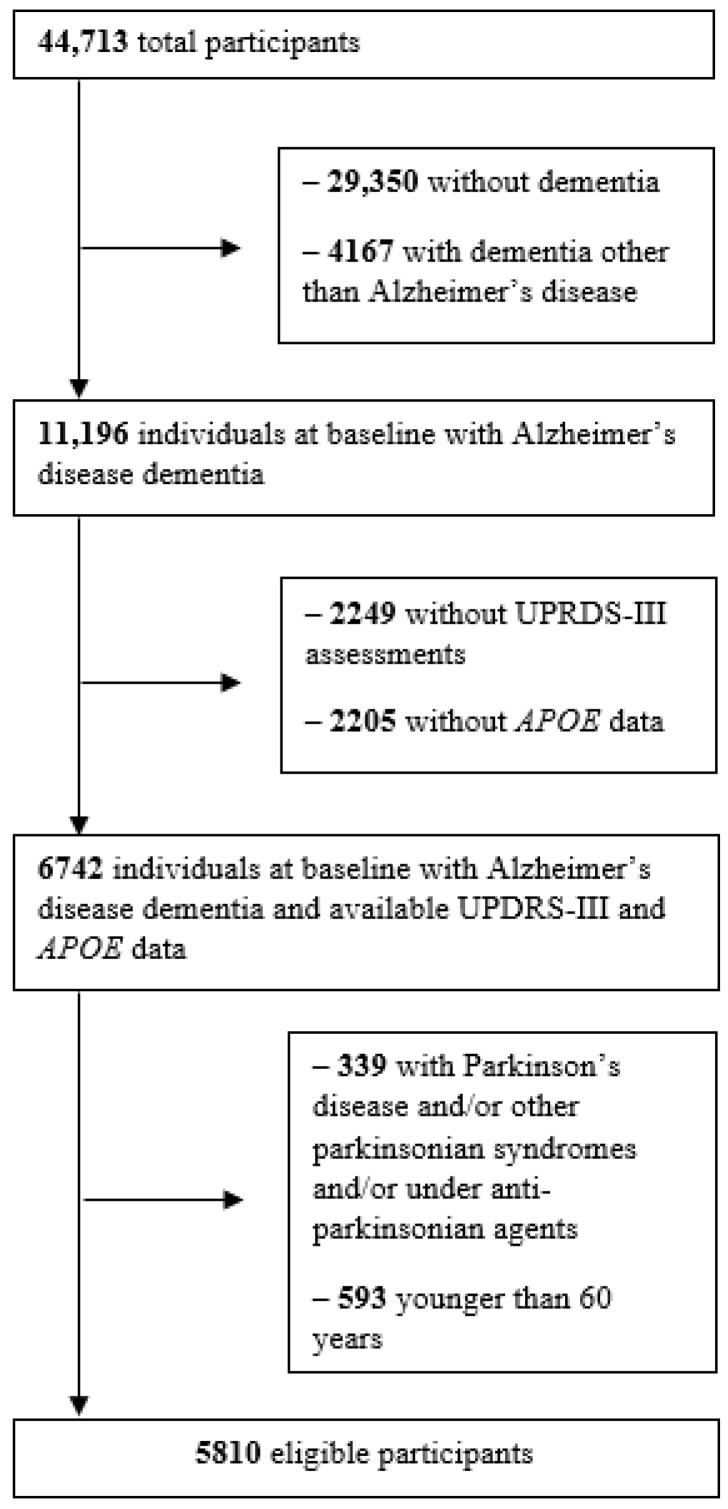
Participant flowchart. *APOE: apolipoprotein E*; UPDRS-III: Unified Parkinson’s disease rating scale part III.

The starting database included 44,713 individuals with at least one UDS evaluation. Among them, 15,363 were diagnosed with dementia and 11,196 with AD. Motor symptoms were assessed using the UPDRS-III only in the first two versions of the UDS; thus, 2249 participants evaluated on the 3rd version were excluded. Data on *APOE* were available for 6742 of the 8947 participants with AD and UPDRS-III assessments. After exclusion of those with a diagnosis of PD or other parkinsonian syndrome, those on anti-parkinsonian medication, and those under the age of 60 years, 5810 participants were eligible for the present study (Figure 1).

Among eligible participants, 831 were missing data on at least one of the covariates (age, sex, race, education, MMSE, CDR, GDS and/or NPS) and were excluded from our analysis. Individuals without missing data (*N* = 4979) were more often Caucasian (*p* < 0.001) and male (*p =* 0.001) compared to those without missing data (*N* = 831). Action-postural tremor was more common in the former group (*p =* 0.049), whereas the remaining of the motor symptoms were more prevalent in the latter (*p* < 0.001). Those with missing data were older (*p =* 0.010), less educated (*p* < 0.001), performed worse on cognitive testing (MMSE, *p* < 0.001), had greater depression scores (GDS, *p =* 0.001), higher neuropsychiatric burden (NPS, *p* < 0.001) and greater disease severity (CDR, *p* < 0.001). There was no difference in terms of *APOE* genotypic distribution (*p =* 0.152). Among eligible participants without missing data on covariates, none had missing data on resting tremor and rigidity, 2 had missing data on hypophonia, masked facies, action-postural tremor and/or bradykinesia, 22 on impaired posture-gait, 33 on impaired chair rise and 131 on postural instability. Therefore, slightly different participant subsets were analyzed per motor domain.

Participant characteristics by *APOE* group are in Table 1. The *APOE4* group was younger and better educated compared to the other groups. The *APOE2* group had more participants of African American ancestry and fewer of Caucasian ancestry compared to the other groups. Sex distributions were similar among groups. Regarding clinical parameters, the *APOE4* group had lower GDS scores compared to both other groups and performed worse on MMSE compared to the *APOE2* group. The *APOE2* group had lower global CDR scores than the others. NPS distributions were similar among groups. As for most motor manifestations, a trend towards greater prevalence in the *APOE2* group and lower prevalence in the *APOE4* groups was documented (comparisons were significant in the context of rigidity, bradykinesia, impaired chair rise, impaired posture-gait and the global motor variable).

### 2.2. APOE Alleles and Motor Signs in Older Adults with AD

Crude binary logistic regression models revealed that compared to the *APOE2* group, the *APOE4* group exhibited lower odds having at least one motor sign [OR = 0.64, 95% CI = (0.50, 0.80)] (Table 2). Significant findings related to rigidity (OR = 0.53), bradykinesia (OR = 0.56), impaired chair rise (OR = 0.54) and impaired posture gait (OR = 0.54). Adjusted models confirmed these findings (Table 2). Intermediate differences in terms of effect size were found between the *APOE2* and *APOE3* groups.

**Figure 2:**
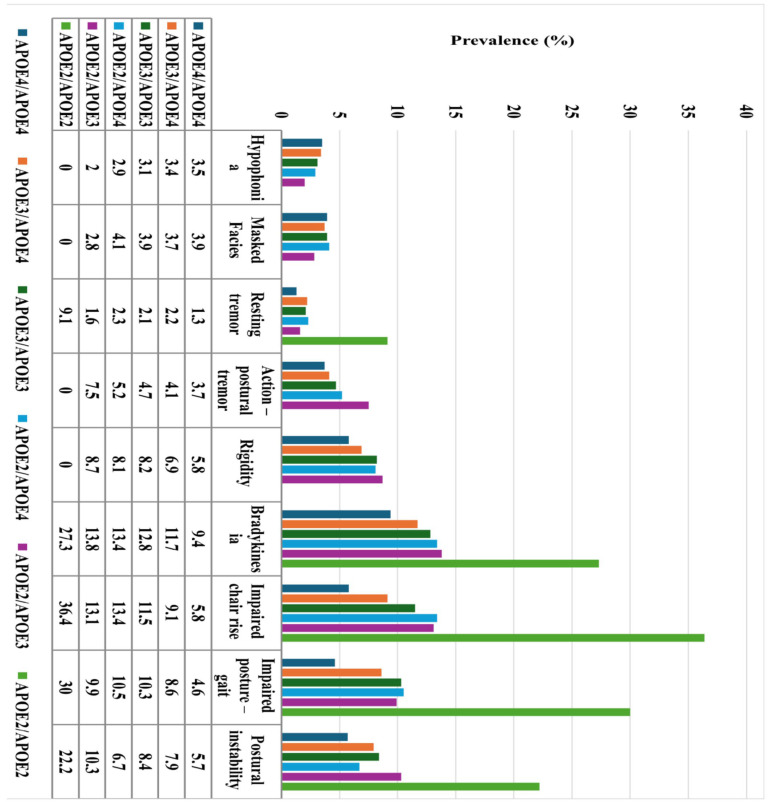
Prevalence [(those with the respective motor sign per genotype/those with the genotype) *100] of motor manifestations per apolipoprotein E (*APOE*) genotype in older adults with Alzheimer’s dementia. Different APOE genotypes are represented by different colors (see annotation). The prevalence (%) of each motor sign by *APOE* genotype is also noted in the table of contents below the bar chart.

Figure 2 shows the prevalence of motor manifestations by *APOE* group. Except for hypophonia, masked facies and resting tremor, there is a clear trend towards more frequent motor manifestations among *APOE2* carriers while less frequent in *APOE4* carriers, suggesting a dose–response pattern for both alleles. Exploratory analyses captured this dose–response relationship for bradykinesia, rigidity, impaired posture-gait and impaired chair rise (Table 3). Specifically, the *APOE2/APOE2* genotype was more closely associated with these signs than *APOE3/APOE2*. The latter exhibited more prominent associations than *APOE4/APOE2*. Sequentially, *APOE3/APOE4* and *APOE4/APOE4* genotypes exhibited relatively (compared to *APOE3/APOE3*) protective properties in an *APOE4* dose-dependent fashion. Overall, *APOE2* seemed to enlarge and *APOE4* to moderate the risk of these motor signs. On the other hand, the aforementioned pattern was not apparent in the remaining motor signs (with the potential exception of hypophonia).

## 3. Discussion

Our purpose was to investigate associations between *APOE* genotype and motor manifestations in AD. Compared to the *APOE4* allele, the *APOE2* allele confers a greater risk for motor signs in older adults with AD. These findings were most evident for bradykinesia, rigidity, impaired posture-gait and impaired chair rise. Intermediate in terms of size associations were found for the comparison of *APOE2* versus *APOE3*. The consistency (effect sizes and directions) and level of certainty (*p*-values) of these associations (*p* < 0.05 for global motor variable, bradykinesia, impaired posture-gait, impaired chair rise) were indicative of non-trivial differences between *APOE2* and *APOE3* (smaller than the difference between *APOE2* and *APOE4*). Exploratory analyses of *APOE* genotypes suggested a dose–response relationship for both *APOE2* (risk-conferring properties) and *APOE4* (protective properties) with respect to motor signs.

While the risk-conferring effect of *APOE4* carriage towards major neurocognitive disorders such as AD, Dementia with Lewy Bodies and Parkinson’s Disease Dementia, is well-established [16], the exact role of *APOE2* carriage in neurodegeneration is less clear. An overall protective effect has been reported for *APOE2* against several dementias [9,10,26,27]. On the other hand, a risk-conferring effect for *APOE2* towards PD has been reported [17,18]. Our findings concur that motor signs, especially parkinsonian manifestations (bradykinesia, rigidity, impaired posture-gait) in AD may be driven by *APOE2*. However, the pathophysiologic mechanisms behind this relationship remain unclear.

*APOE2* may protect against β-amyloid accumulation, the neuropathological hallmark of AD [28]. It is also associated with reduced global tau deposition in the AD continuum especially within the medial temporal regions, the earliest regions to display AD-related neurodegenerative alterations [29,30]. On the other hand, *APOE2* has been associated with primary tauopathies (e.g., progressive supranuclear palsy, argyrophilic grain disease) and more pronounced tau depositions in these entities [11,15,31]. Collectively, these findings suggest that APOE2 mitigates secondary tau deposition (as in AD) but drives primary tau pathology [9]. Hence, the relationship between *APOE2* and motor signs in AD may be related to the different neuropathological alterations that accompany this allele. Less prominent β-amyloid pathological changes, less pronounced tau deposition in the entorhinal cortex and greater primary tau pathology, may explain the increased prevalence of motor signs associated with *APOE2*.

The association between *APOE2* and cerebral amyloid angiopathy (CAA) might provide an alternative explanation (to increased primary tau pathology). Although *APOE2* protects against plaque Aβ, it is related to increased CAA [32,33]. Despite the intersections of vascular and plaque Aβ, vascular pathology is more probable to disrupt motor pathways and contribute to parkinsonian features. Decreased basal ganglia volumes, cerebral white matter and subcortical cerebellar atrophy, have been correlated with CAA and may be among the pathological pathways that mediate the occurrence of motor manifestations in CAA [34,35,36].

Finally, it is possible that the association between *APOE2* and motor signs is related to the milder clinical course of AD. Individuals with *APOE2* and AD have an overall better cognitive and functional status as well as prolonged survival compared to those with *APOE4* and AD [37]. Therefore, in the long term, motor symptoms and signs that would otherwise go unnoticed may be unmasked.

This study has several strengths including the large sample of genotyped older individuals with AD. The extensive characterization in the UDS allowed us to account for many important confounders: demographics, cognitive performance, depression scores, neuropsychiatric burden, AD stage [32]. Nevertheless, the analysis has several weaknesses as well, including being cross-sectional, whereas causality assumptions are strengthened by longitudinal associations. Of course, genetic data are often analyzed using cross-sectional and case–control study designs (since genetic exposures always precede phenotypes investigated). Even in this case, however, statistical associations are not equivalent to causal associations. Population stratification and linkage disequilibrium can lead to spurious results. However, longitudinal designs do not account for population stratifications (inherent between group ancestry differences in both genetics and outcomes investigated) or linkage disequilibrium (non-random co-inheritance patterns). Therefore, future studies using mendelian randomization ought to confirm our findings. Second, although several important covariates were considered, our findings may have been driven by residual confounding or non-trivial proportion of missing data. Further, in most cases, the diagnosis of AD and other dementias was based on clinical criteria; biomarkers were not uniformly available. Therefore, there may have been misclassification of other neurodegenerative conditions such as AD. Future investigations should incorporate cases with biomarker-supported clinical diagnoses to improve diagnostic accuracy and avoid the potential misclassification of dementia cases. Additionally, biomarker investigations (blood, cerebrospinal fluid and imaging studies) may enhance our understanding of the intrinsic connections between APOE genotypes, neuropathology and motor signs in older adults with AD (e.g., to shed light onto the potential presence of alternative non-AD neuropathologies that mediate the presence of motor signs in AD). Next, although UPDRS-III is widely used in both clinical and research settings, variability can be expected across different examiners in quantifying motor signs. To overcome this shortcoming, improve the reliability of motor assessments and quantify the severity of the symptoms, future studies may incorporate instrumented tools instead of UPDRS-III measurements. Tapping tasks with keyboards for bradykinesia, accelerometers/gyroscopes for tremor, posturography for balance, as well as other wearable monitors and kinematic sensors may be utilized for this purpose. Finally, the count of some motor signs (hypophonia, resting tremor, masked facies) was very small, leading to lack of power in analyses involving these signs.

## 4. Materials and Methods

We analyzed cross-sectional UDS data for associations between *APOE* alleles and motor signs in older adults with AD. The UDS assembles standardized, prospectively collected, multidisciplinary data from multiple National Institute on Aging/National Institutes of Health (NIA/NIH)—funded Alzheimer’s Disease Centers (ADCs) across the United States. The UDS is freely available to research scientists upon request (https://naccdata.org/). All study procedures were approved by the Institutional Review Boards overseeing each ADC prior to the initiation of the study. All participants granted informed consent prior to participation to the study. The rationale and the key methodological features of the UDS have been detailed elsewhere [38,39,40,41]. Briefly, each participating ADC enrolls volunteers with a cognitive status ranging from normal cognition to dementia. Participants may actively pursue professional consultation, may be referred to an ADC by other clinicians or family members, may be actively recruited, etc., according to the protocol of each ADC. Trained physicians and clinic personnel evaluate participants on an approximately yearly basis using a uniform, standardized protocol. Although the focus of the UDS is AD, data are also collected on other neurocognitive and neuropsychiatric disorders.

### 4.1. Eligibility Criteria and Diagnostic Procedure

The present study was based on cross-sectional NACC data from the inception of the UDS (September 2005) to the December 2022 data freeze. Data from a total of 46 ADCs were involved. We focused on baseline evaluations of older adults, over 60 years old, with a diagnosis of AD but without a concomitant diagnosis of PD or other parkinsonian syndrome. Participants being treated with anti-parkinsonian medications were excluded. Depending on the specific protocol of each ADC, cognitive diagnoses were established by either an interdisciplinary consensus team (in most cases) or a single clinician (who examined the participant). Diagnoses were based on extensive standardized evaluations including personal and family medical history, clinical examinations, neuropsychological and neuropsychiatric assessments, psychosocial functioning evaluations, and so on. Dementia was diagnosed using standard clinical criteria [42,43,44,45]. Imaging and/or cerebrospinal fluid biomarkers were only available in a minority of cases [46].

### 4.2. Measurement of Motor Signs Based on the UPDRS-III

The Unified Parkinson’s disease rating scale part III (UPDRS-III), which was administrated in the first two versions of the UDS, comprises 27 subitems. We grouped these into 9 domains as follows: (1) hypophonia (single item); (2) masked facies (single item); (3) resting tremor (combined five items regarding tremor at rest in the face/lips/chin and four extremities); (4) action/postural tremor (combined two items regarding tremor at rest in the hands); (5) rigidity (combined five items regarding rigidity in the neck and four extremities); (6) bradykinesia (combined nine items: bilateral finger tapping, hand movements, rapid alternating movements of the hands, leg agility, and body bradykinesia); (7) impaired chair rise (single item); (8) impaired posture/gait (combined two items: posture and gait); and (9) postural instability (single item).

Each motor subitem was graded as absent (score < 2) or present (score ≥ 2). The rationale for this cutoff is as follows: (1) this severity (≥2) is more likely to be noted by the average clinician [47]; and (2) a score of 1 is suggestive of a very mild motor change that could be observed with normal aging [48]. Then, we created nine dichotomous variables (motor domains), such that participants were said to have an abnormal motor sign if they scored ≥2 in at least one of the subitems of the respective motor domain [49]. A global dichotomous variable was also created (presence of at least one motor sign), according to which participants were divided into those with (if they scored ≥2 in at least one motor domain) and those without (if they scored <2 in all motor domains) any motor manifestations.

### 4.3. Apolipoprotein E Genotyping and Grouping

*APOE* haplotypes for NACC were determined from the single-nucleotide variants *rs7412* (*APOE2*) and *rs42935848* (*APOE4*). We clustered participants into three groups, based on *APOE* genotypes: *APOE2* (*APOE2/APOE3*, *APOE2/APOE4*, or *APOE2/APOE2*), *APOE3* (*APOE3/APOE3*) or *APOE4* (*APOE3/APOE4* or *APOE4/APOE4*).

### 4.4. Covariates Considered

Age at visit (years), education (years of formal schooling), Mini-Mental State Examination scores (MMSE—cognitive performance) and Geriatric Depression Scale (GDS) scores were treated as scale variables. Sex (male/female), race (Caucasian, African American, Asian, other), global CDR (0.5, 1.0, 2.0, 3.0—cognitive symptom severity) and neuropsychiatric symptom severity (NPS)—no, mild, moderate-severe NPS—were treated as categorical variables.

NPS severity was assessed using data from the Neuropsychiatric Inventory Questionnaire (NPI-Q) [50]. NRI-Q evaluates 12 domains [delusions, hallucinations, agitation/aggression, depression/dysphoria, anxiety, elation/euphoria, apathy/indifference, disinhibition, irritability/lability, aberrant motor behavior, night-time behaviors, and appetite/eating] according to a 4-point severity scale: no, mild (noticeable, but not a significant change); moderate (significant, but not a dramatic change); or severe (very marked or prominent; a dramatic change). For each NPI-Q domain, participants were grouped into three categories: 0: absent; 1: mild; 2: moderate and severe symptomatology (due to the small prevalence of moderate and severe symptoms) [51]. Sequentially, the composite NPS was calculated as follows: 0: no symptoms; 1: at least one mild symptom with no moderate and/or severe symptoms, 2: at least one moderate and/or severe symptom.

### 4.5. Statistical Analysis

Differences between the three *APOE* groups were analyzed using (1) analysis of variance with Bonferroni correction (scale variables) and (2) Pearson’s chi-squared tests (categorical variables). Associations between the three *APOE* groups and motor signs were estimated using separate (for each motor domain) binary logistic regression models (main analysis). Both crude and adjusted models were tested. Adjusted models featured the above set of covariates. Followingly, an exploratory analysis featuring the six individual *APOE* genotypes was performed, to assess whether a dose–response relationship existed (increased magnitude of effect sizes with homozygosity compared to heterozygosity). The exploratory analysis was identical to the main analysis (adjusted for the same covariates) except for the *APOE* variable, which featured the six *APOE* genotypes instead of the three *APOE* groups.

The analyses were performed using the IBM SPSS Statistics Software Version 27 (Chicago, IL, USA). The conventional threshold of α = 0.05 was implemented to assess for statistical significance in baseline comparisons. The stricter α = 0.005 cut-point, corrected for multiple (*N* = 10) comparisons, was used in the main analysis. Effect sizes (odds ratios, ORs) and precision estimates (95% confidence intervals, 95% CIs) are provided.

## 5. Conclusions

Compared to *APOE4, APOE2* confers an increased risk towards motor signs, including bradykinesia, rigidity, impaired posture-gait and impaired chair rise, in older adults with AD. *APOE3* may confer an intermediate risk. A dose–response relationship for both *APOE2* (risk-conferring properties) and *APOE4* (protective properties) alleles may exist. To explain these associations, future research applying more sophisticated imaging and/or post-mortem investigations should explore pathologic changes in older APOE2 carriers with AD and motor signs. Our findings may have clinical implications for phenotypic subgroup definition and precision medicine. For instance, genotype-informed diagnostic frameworks may facilitate the diagnosis of complex AD phenotypes (e.g., with parkinsonian features). By extension, future studies may look into the relationship between APOE status and biomarker profiles (e.g., tau distribution, CAA) and reveal new associations. Regardless, considering the phenotypic associations, clinical trials in AD may be appropriate to stratify participants by APOE status and even consider motor endpoints for APOE2 carriers.

## Figures and Tables

**Table 1:** Participant characteristics per *APOE* group.

Variable	*APOE2* (N = 389)	*APOE3* (N = 1799)	*APOE4* (N = 2791)	*p*-Value
Age (years)	77.6 ± 8.1	77.9 ± 8.3	75.3 ± 7.4	*p <* 0.001
Sex (male/female)	176/213 (45.2/54.8)	850/949 (47.2/52.8)	1227/1564 (44.0/56.0)	*p =* 0.092
Education (years)	13.9 ± 3.5	14.0 ± 3.9	14.4 ± 3.5	*p =* 0.001
Race (Caucasian/African American/Asian/other)	316/60/4/9 (81.2/15.4/1.0/2.3)	1519/181/29/70 (84.4/10.1/1.6/3.9)	2360/356/26/49 (84.6/12.8/0.9/1.8)	*p* < 0.001
MMSE (30)	21.63 ± 4.8	21.0 ± 5.5	20.8 ± 5.4	*p =* 0.018
GDS (15)	2.8 ± 2.8	2.6 ± 2.6	2.4 ± 2.5	*p =* 0.001
NPS (none/mild/moderate or severe)	73/127/189 (18.8/32.6/48.6)	279/601/919 (15.5/33.4/51.1)	456/973/1362 (16.3/34.9/48.8)	*p =* 0.362
Global CDR (0.5/1.0/2.0/3.0)	151/196/41/1 (38.8/50.4/10.5/0.3)	605/868/279/47 (33.6/48.2/15.5/2.6)	959/1357/415/60 (34.4/48.6/14.9/2.1)	*p =* 0.012
Global motor variable (No/Yes)	266/123 (68.4/31.6)	1277/522 (71.0/29.0)	2157/634 (77.3/22.7)	*p* < 0.001
Hypophonia (No/Yes)	383/6 (98.5/1.5)	1770/27 (98.5/1.5)	2753/38 (98.6/1.4)	*p =* 0.907
Masked Facies (No/Yes)	379/10 (97.4/2.6)	1762/36 (98.0/2.0)	2738/52 (98.1/1.9)	*p =* 0.638
Resting tremor (No/Yes)	382/7 (98.2/1.8)	1768/31 (98.3/1.7)	2747/44 (98.4/1.6)	*p =* 0.902
Action–postural tremor (No/Yes)	362/27 (93.1/6.9)	1713/85 (95.3/4.7)	2671/119 (95.7/4.3)	*p =* 0.062
Rigidity (No/Yes)	360/29 (92.5/7.5)	1681/118 (93.4/6.6)	2655/126 (95.5/4.5)	*p =* 0.002
Bradykinesia (No/Yes)	337/52 (86.6/13.4)	1603/196 (89.1/10.9)	2546/243 (91.3/8.7)	*p =* 0.003
Impaired chair rise (No/Yes)	341/46 (88.1/11.9)	1606/181 (89.9/10.1)	2601/171 (93.8/6.2)	*p* < 0.001
Impaired posture – gait (No/Yes)	349/39 (89.9/10.1)	1635/157 (91.2/8.8)	2628/149 (94.6/5.4)	*p* < 0.001
Postural instability (No/Yes)	342/30 (91.9/8.1)	1613/129 (92.6/7.4)	2563/171 (93.7/6.3)	*p =* 0.199

Scale variables are presented as mean ± standard deviation; categorical variables are presented as absolute numbers (proportions); *p*-value corresponds to among group differences; N: number of individuals; *APOE*: *apolipoprotein E*; MMSE: mini-mental state examination; GDS: geriatric depression scale; NPS: neuropsychiatric score; CDR: clinical dementia rating scale.

**Table 2:** Associations between *APOE* alleles and motor manifestations in older adults with Alzheimer’s dementia.

Variable (Omnibus p)	*APOE4* Versus *APOE2*	*APOE3* Versus *APOE2*
Crude		
**Global motor variable (*p* < 0.001)**	0.64 (0.50, 0.80), *p* < 0.001	0.88 (0.70, 1.12), *p =* 0.307
Hypophonia (*p =* 0.907)	0.88 (0.37, 2.10), *p =* 0.775	0.97 (0.40, 2.38), *p =* 0.953
Masked Facies (*p =* 0.640)	0.72 (0.36, 1.43), *p =* 0.347	0.77 (0.38, 1.57), *p =* 0.480
Resting tremor (*p* = 0.902)	0.87 (0.39, 1.95), *p =* 0.743	0.95 (0.42, 2.19), *p =* 0.917
Action – postural tremor (*p =* 0.065)	0.60 (0.39, 0.92), *p =* 0.019	0.67 (0.43, 1.04), *p =* 0.074
**Rigidity (*p =* 0.003)**	0.59 (0.39, 0.89), *p =* 0.013	0.87 (0.57, 1.33), *p =* 0.522
**Bradykinesia (*p =* 0.003)**	0.62 (0.45, 0.85), *p =* 0.003	0.79 (0.57, 1.10), *p =* 0.164
**Impaired chair rise (*p* < 0.001)**	0.49 (0.35, 0.69), *p* < 0.001	0.84 (0.59, 1.18), *p =* 0.306
**Impaired posture – gait (*p* < 0.001)**	0.51 (0.35, 0.73), *p* < 0.001	0.86 (0.59, 1.24), *p =* 0.421
Postural instability (*p =* 0.200)	0.76 (0.51, 1.14), *p =* 0.184	0.91 (0.60, 1.38), *p =* 0.662
Adjusted		
**Global motor variable (*p =* 0.001)**	0.64 (0.50, 0.82), *p* < 0.001	0.75 (0.59, 0.97), *p =* 0.027
Hypophonia (*p =* 0.623)	0.64 (0.26, 1.56), *p =* 0.331	0.67 (0.27, 1.71), *p =* 0.404
Masked Facies (*p =* 0.283)	0.58 (0.29, 1.16), *p =* 0.127	0.58 (0.28, 1.17), *p =* 0.136
Resting tremor (*p* = 0.903)	0.83 (0.37, 1.87), *p =* 0.652	0.85 (0.37, 1.96), *p =* 0.699
Action – postural tremor (*p =* 0.047)	0.58 (0.38, 0.91), *p =* 0.017	0.59 (0.38, 0.93), *p =* 0.024
**Rigidity (*p =* 0.004)**	0.53 (0.34, 0.81), *p =* 0.004	0.74 (0.48, 1.14), *p =* 0.176
**Bradykinesia (*p =* 0.002)**	0.56 (0.40, 0.77), *p =* 0.001	0.65 (0.46, 0.91), *p =* 0.013
**Impaired chair rise (*p =* 0.003)**	0.54 (0.37, 0.78), *p =* 0.001	0.69 (0.47, 0.99), *p =* 0.046
**Impaired posture – gait (*p =* 0.008)**	0.54 (0.36, 0.81), *p =* 0.003	0.67 (0.45, 0.99), *p =* 0.046
Postural instability (*p =* 0.237)	0.88 (0.57, 1.34), *p =* 0.545	0.73 (0.47, 1.13), *p =* 0.163

**Bold** denotes statistical significance; *APOE*: *apolipoprotein E*; effect sizes and precision estimates represent odds ratios and 95% confidence intervals; odds ratios lower than 1 are consistent with a risk effect for *APOE2* (protective effect for *APOE4* or *APOE3*); between group differences were considered significant only if among group differences (omnibus p) were significant at the stricter-corrected *p*-value of α = 0.005.

**Table 3:** Associations between *APOE* genotypes and motor manifestations in older adults with Alzheimer’s dementia. Τhe *APOE3*/*APOE3* genotype was used as reference, i.e., odds ratios greater than 1 represent risk effects for the respective genotypes, whereas odds ratios lower than 1 represent protective effects (when compared with the *APOE3/APOE3* genotype).

Variable	*APOE4*/ *APOE4*	*APOE3*/ *APOE4*	*APOE4*/ *APOE2*	*APOE3*/ *APOE2*	*APOE2*/ *APOE2*
Global motor variable (*p =* 0.003)	0.88 (0.70–1.12) (*p =* 0.299)	0.84 (0.72–0.98) (*p =* 0.029)	1.01 (0.68–1.50) (*p =* 0.969)	1.57 (1.16–2.12) (*p =* 0.004)	1.12 (0.23–5.37) (*p =* 0.888)
Hypophonia (*p =* 0.906)	0.75 (0.31–1.79) (*p =* 0.512)	1.01 (0.58–1.73) (*p =* 0.982)	1.39 (0.32–6.12) (*p =* 0.662)	1.62 (0.53–4.91) (*p =* 0.397)	N/A
Masked Facies (*p =* 0.706)	0.92 (0.45–1.86) (*p =* 0.813)	1.03 (0.65–1.65) (*p =* 0.893)	1.89 (0.65–5.49) (*p =* 0.240)	1.74 (0.71–4.25) (*p =* 0.225)	N/A
Resting tremor (*p* = 0.933)	0.69 (0.30–1.61) (*p =* 0.393)	1.05 (0.65–1.72) (*p =* 0.833)	1.34 (0.40–4.47) (*p =* 0.637)	1.14 (0.40–3.29) (*p =* 0.807)	N/A
Action – postural tremor (*p =* 0.206)	0.98 (0.62–1.54) (*p =* 0.924)	0.99 (0.73–1.34) (*p =* 0.943)	1.54 (0.75–3.16) (*p =* 0.236)	1.88 (1.10–3.22) (*p =* 0.021)	N/A
Rigidity (*p =* 0.013)	0.62 (0.40–0.98) (*p =* 0.041)	0.74 (0.56–0.98) (*p =* 0.034)	1.00 (0.47–2.11) (*p =* 0.999)	1.66 (1.01–2.74) (*p =* 0.045)	N/A
Bradykinesia (*p =* 0.006)	0.74 (0.52–1.05) (*p =* 0.094)	0.88 (0.71–1.10) (*p =* 0.259)	1.21 (0.69–2.10) (*p =* 0.505)	1.67 (1.12–2.51) (*p =* 0.015)	4.12 (0.93–18.27) (*p =* 0.062)
Impaired chair rise (*p =* 0.029)	0.89 (0.58–1.35) (*p =* 0.571)	0.77 (0.60–0.98) (*p =* 0.037)	1.28 (0.71–2.32) (*p =* 0.409)	1.55 (0.99–2.43) (*p =* 0.058)	2.02 (0.32–12.80) (*p =* 0.455)
Impaired posture – gait (*p =* 0.014)	0.64 (0.39–1.06) (*p =* 0.082)	0.85 (0.65–1.10) (*p =* 0.204)	1.15 (0.59–2.26) (*p =* 0.677)	1.59 (0.99–2.55) (*p =* 0.058)	6.40 (1.10–37.26) (*p =* 0.039)
Postural instability (*p =* 0.349)	1.43 (0.93–2.18) (*p =* 0.102)	1.15 (0.88–1.50) (*p =* 0.293)	0.95 (0.45–2.04) (*p =* 0.903)	1.62 (0.97–2.71) (*p =* 0.063)	1.62 (0.16–15.95) (*p =* 0.682)

*APOE*: *apolipoprotein E*; effect sizes represent odds ratios; N/A: non-applicable due to lack of ‘events’.

## Data Availability

For further information on access to the NACC database, please contact NACC (contact details can be found at https://naccdata.org/).

## Abbreviations

The following abbreviations are used in this manuscript:

APOE	apolipoprotein E
AD	Alzheimer’s Disease dementia
PD	Parkinson’s Disease
UDS	Uniform Data Set
NACC	National Alzheimer’s Coordinating Center
NIA/NIH	National Institute on Aging/National Institutes of Health
ADCs	Alzheimer’s Disease Centers
UPDRS-III	Unified Parkinson’s Disease Rating Scale part III
MMSE	Mini-Mental State Examination
GDS	Geriatric Depression Scale
CDR	Clinical Dementia Rating
NPS	Neuropsychiatric Symptom Severity
NPI-Q	Neuropsychiatric Inventory Questionnaire
ORs	Odds Ratios
95% CIs	95% Confidence Intervals
